# Injurious Effects of Tight Lacing

**Published:** 1830-04-01

**Authors:** 


### XXXV.

### INJURIOUS EFFECTS OF TIGHT LACING. By DR. GODMAN.

"It is not without hesitation that the writer ventures to call attention to the injuries produced by TIGHT LACING, being well aware that he is exposing himself to the chance of severe animadversion for appearing to meddle officiously with the concerns of the fair sex, who never fail to punish every encroacher upon their rights and privileges. Notwithstanding, as our object is, if possible, to avert great suffering and much future misery, by setting forth the evils following manifest abuses, introduced and augmented by fashion, we hope due indulgence will be extended by our fair readers, whose real good we are most solicitous to promote.

The observations of various authors have satisfactorily shown, that certain errors in dress and exercise induce deformity of person and unhappiness of mind; but their attention is almost entirely devoted to the injuries done to the organs of support and motion, the bones and muscles.* Great as are the evils they treat of, they seem slight when compared with the pernicious effects of similar causes, on organs more immediately essential to the life of the individual, the disarray of which, though not signalized by very obvious deformity, is inevitably followed by protracted debility and suffering, an early, rapid decay, or a painful and premature death. It is impossible for a benevolent mind, acquainted with the reality and extent of the mischief thus produced, to behold youth, grace and

* See the works of Shaw, Duffin, &c., on Deformities of the Spine, &c.

beauty sacrificing the dearest boons of life to the tyranny of perverted taste and preposterous fashion, without experiencing emotions of profound regret for the immediate victims, and sighing for the future condition of a posterity derived from such a parentage !

In what way can the hitherto irresistible torrent of fashion be stemmed ? Have not reason and experience been appealed to in vain ? Have not the shafts of satire, the serious remonstrances of morality, and even the awe-inspiring declarations of religion, too often fallen ineffectual to the ground ? One mode of producing the desired conviction in the minds of females, has been left almost unattempted ; and from the operation of this method much is to be hoped. It is by imparting to "nature's last, best work," a sufficient knowledge of the peculiar construction of the human system, to place in the clearest light the dreadful risks those run who indulge in the vices of dress, and the cruel maladies which are certainly induced in delicate frames by such as persist in disregarding the warnings offered by reason and science. To us it appears scarcely possible that a female of ordinary intelligence can become even superficially acquainted with the curious actions necessary to the processes of breathing, circulation, and nutrition, without shrinking in terror at the thought of the dangers to which those are exposed, who intentionally counteract nature in all her benevolent designs, by violently compressing their persons, according to whatever model capricious and ever-varying fashion may dictate.

That part of the human frame most immediately subjected to tight lacing, is not only one of its most lovely external proportions, but contains and defends the organs so important and indispensable to existence, the LUNGS and HEART, which perform the functions of respiration and circulation, to purify and perfect the blood, and send its rich and vivifying streams to the remotest extremities of the system. On the perfect action of these great organs depend all our vigor and elasticity ; the roseate bloom and radiant eye of beauty ; the joyous buoyancy of youth, and the steady sereneness of maturity. When these functions are impaired, pallid features, anguishing debilities, melancholy depression of spirits, agonizing decay, and a long train of ghastly maladies, destructive of hope, and rendering life a burthen, must necessarily ensue.

The part of our structure to which allusion is made, is popularly called "the chest ;" and to judge them by their practice, many of our fair country-women regard it as a mere empty, flexible case, which may safely be squeezed into whatever compass the possessor pleases. Unfortunately for them, this is far from the reality ; the chest is an admirably complex contrivance, whose free motions are as necessary to breathing and circulation, as these processes are to health and life. Consequently, whatever diminishes the capacity of the chest, proves directly injurious by excluding the air, and every impediment to its movements prevents the proper transmission of the blood through the lungs.

As all the parts described [those concerned in forming and filling up the chest] are flexible and moveable from their peculiar nature and connexions, it is obvious that the first effect of any tightness or constriction will be to impede their proper motions, and thrust them out of their natural position. Thus, the corset being laced tightest at the part of the chest having the shortest ribs, the longest and most flexible cartilages, and the most extensive motion, produces narrowing of the chest, renders its free movements impossible, and permanently deforms it by doubling the cartilages inward near their junction with the breast bone. As if this mischief were not great enough, another instrument of torture is added, in the form of a steel or hictory *busk*, which is pushed into its sheath in the already too tight corset, immediately over, and extending along, the whole length of the breast bone. This busk is to keep the body from bending forward in the centre, and to prevent the dress and corset from 'hooping up,' as it is called

As the body cannot possibly be prevented from leaning forward to a certain degree, the consequence is, that the whole weight of the superior part is sustained upon the lower part of the breast-bone, which leans directly against the busk, at a point where it is least supported by the attachment of the cartilages of the ribs. The point thus injuriously pressed upon, is nearly opposite the lesser end of the stomach, and most of those who habitually lace tight, have a depression here which would contain the size of half an egg. Either a constant feeling of aching and soreness is experienced at this point, or when the busk is taken out, it is so sore and painful that the individual cannot bear the slightest pressure without an exclamation of distress.

We have, then, among the first effects of the tight lacing and pressure of the busk, impairment of motion and deformity of the chest, accompanied by a constant soreness and irritation over the stomach, whose undisturbed action is one of the greatest essentials to health. If, however, this was the sum of the evil, we might regard it as tolerable, being apparently external. But when the lower part of the chest is compressed, the liver is by the same force squeezed upwards and inwards, and, being a large and solid body, it pushes before it the diaphragm, and forcibly prevents its descent in the act of breathing; while on the other side, the spleen and stomach are forced upwards, producing a similar effect on the diaphragm; and the functions of all these organs, the liver, stomach, and spleen, must be impaired in proportion to the pressure and displacement their delicate nerves and vessels suffer. In addition to these greater or more obvious injuries to the functions of individual organs, we may now add the evils caused to the great vital functions. The same pressure which forces the liver, &c. inwards and upwards, by squeezing the texture of the organs together, prevents the free entrance of the blood into them, and by being thrust firmly back against the spine and lower part of the diaphragm, they compress the openings by which the blood passes to and from the heart, through the great vein and artery. The consequence of thus damming up the vital current, is the gradual development of irregularity of action in the heart, palpitations, tendency to faint, violent throbbings, and in some cases organic alteration in the heart itself. This same tightening of the lower part of the chest, and prevention of the enlargement of its cavity by stopping the descent of the diaphragm, acts with equal injury on the blood which should descend from the great veins of the head and arms to the heart at each breathing. The proper quantity of blood cannot be delivered therefrom, for want of proper dilatation of the chest, and the individual is subject to violent headachs, dulness, low spirits, extreme paleness, or leaden hue of countenance.

These readily observable consequences are but the commencement of ills from this source. The lungs being withheld from their proper actions by not being sufficiently dilated, the air cannot get access to the blood, and the blood cannot receive that purification or elaboration which renders it fit to sustain the body in health. Its watery, carbonaceous, and other impurities, are retained instead of being thrown off, and in place of a brilliant vermilion-colored fluid being sent to the left side of the heart for the general system, it returns of a dark or bluish red, scarcely better than when it entered the lungs, and almost utterly unfit for any of the purposes of life. This condition, if kept up, is soon made sensible by defective energy in all parts of the body, by various local diseases, and slight morbid changes, sufficient to render life irksome. Cold extremities, pale visages, troubled sleep, excessive mobility of system, commonly called *nervousness*, evinced by great agitation from very inadequate causes, &c. are among the most generally obvious consequences of such impairments of function.

To say nothing farther of the actual mischiefs which tight lacing produces, the influence it exerts on all the motions of the body is entirely disadvantageous. Can anything on earth be more ungraceful than the gait, the walk of a female who is extremely corsetted? From the shoulders down, as stiffly inflexible as the parlour tongs, she can only advance by a sideling shuffle of the feet, which appear to get forward by stealth, instead of moving the body with that elastic mobility characteristic of its unrestrained natural condition. Instead of the easy graceful inclination of a flexible form, we have an awkward ungainly attempt to balance the body on the limbs; the shoulders stiffened backwards, as if shackled with iron; the chest girded in, till breath can scarcely be drawn; and the trunk of the body as rigid as if carved in wood,—the figure looking like a caricature upon nature, ease, and grace! When ladies in this trim enter a room, especially after walking, they can scarcely speak for several minutes, and their bosoms heave with an unnatural agitation. If the busk be of the *fashionable* length, it is impossible for them to sit comfortably in a chair; they must perch on its outer edge, to prevent the busk from being pushed towards the chin, &c. All this torture, uneasiness, and inconvenience, is patiently endured, and for what? because it is fashionable! Grace, ease, elegance, and comfort, are alike immolated to this Moloch, who spares none who pretend to the rank of *fashionable!*

In persons of somewhat more robust frames, the use of tight corsets is followed by a very severe pain, which is experienced at the time of taking them off, and rather different in kind from that we have mentioned as occurring to delicate females. The pain in this case is caused by the return of the blood to the parts which have been violently compressed by the corsets, and enjoyed but a partial circulation while they were worn. It is exceedingly acute, and requires the corset to be very gradually loosened. Some idea of it may be formed by those who have occasionally taken off a very tight garter or other ligature, which has been worn for some hours. We feel less commiseration for such sufferers, who have not the shadow of excuse which is offered by the delicate; they do not need support, and are merely solicitous to make "a figure!"

Very probably it may be urged that the evils we have indicated are confined to a comparatively small number, and that a much greater proportion of females wear corsets without suffering these inconveniences or injuries. However true it may be that some persons use corsets with impunity, it does not in the least diminish the force of the well-founded objections made to them in the preceding observations: it may be said with equal truth, that numerous individuals use spirituous liquors, or amuse themselves by occasional gaming, without injury; yet we know that the vast majority of mankind are but too prone to pass from the use to the abuse of both the latter; and as in the case of spirituous liquors, the transition from the use to the abuse is frequently so gradual as to be nearly imperceptible until the severest evils are produced, so it is most probab'e, especially in young persons, that the use of corsets and busk will speedily and imperceptibly advance to their abuse. There is one circumstance, moreover, which should be particularly remembered, which is, that although ladies properly educated, and aware of the danger of misusing corsets, might employ them without especial injury, the females of lower ranks in life, who imitate what they *see* in those above them, without reference to cause or consequence, will almost inevitably be led to do themselves the worst injuries. We see daily confirmation of this in the attempts of female attendants, &c. to imitate their employers, in the article of *lacing* at least, nor is it at all uncommon for such young women to be obliged to consult physicians for various supposed diseases, which are the immediate results of their preposterous attempts to make themselves "fine figures." Many of them, with this view, keep on their corsets and busks all night, *tightening*, when they lie down, instead of loosening them, and again in the morning drawing them still closer,—considering every successive half inch in the compression and diminution of the lower part of the chest, as so much "clear gain."* The consequences that speedily follow are, loss of appetite, headach, palpitation, and most of the sufferings already mentioned.

After all our researches, we have not been able to discover the exact origin of this ridiculous and injurious mode of dressing. That in one modification or other it has been employed among Europeans for ages, we have unquestionable proof. The circumstance of its being confined principally to those countries whose moral and religious codes have a common foundation, forces us to conclude that the contrivances of stays, corsets, &c. were designed to *conceal*, as far as possible, the consequences of levity and imprudence. The idea of improving the figure by their use, was originally a mere excuse to cover the *real* object for which they were worn. The disposition to imitate, so common to the human race, favoured the views of the depraved and designing, and multitudes of elegant and innocent women fell into a fashion which promised improvement to their personal charms, while in reality it was productive of their destruction. The same phantom of augmenting attractiveness by their employment, contributed to prolong the illusion to the present time, and as our fashionable females have felt the influence produced on their mothers by this folly, we have now the superadded excuse of need of support, on account of muscular debility, urged for its continuance. It is not a little curious to observe the effect that has been produced on female sentiments, by the operation of this cause. The object being to look slender (graceful is utterly impossible, if the body thus dressed be in motion), all rotundity of person is regarded as vulgar or inelegant, though nature has taken infinite pains to render all living forms round and swelling, both externally and internally. Hence the youthful and unmarried are exceeding desirous, by aid of cord and busk, to look *flat*, and in every sense of the term are successful;—the same horror of rotundity follows them through life, and nothing is so common as to find those who have lived and dressed with an exclusive view to gain husbands, with all the mawkishness of false delicacy, using injurious efforts to conceal their approach to the endearments and respectability of maternity. Far be it from our thoughts to wish that our matrons should, in the slightest degree, abate of their sensitiveness on this or any other subject connected with purity of mind; but a close and somewhat protracted observation has fully convinced us, that, from the cause we have mentioned, and others we dare not

---

* Not long since, the following scene occurred under our notice, at a boarding-house in Philadelphia.—The girl of the house, a tall, good-looking young woman, at the proper time in the afternoon, filled the tea-kettle, and brought it to the kitchen hearth, where she placed it on a bench. To place it over the fire required considerable stooping, and this as it turned out, was impossible to her. Repeated and fruitless were her attempts, by a sort of crouching attitude, to accomplish her object; there was no one present to assist or to relieve her from the restraint which prevented stooping, and at length in despair she gave up her trials, and stood by the kettle as if debating what she should do. The mistress came to inquire if the water was boiling, and found it not yet on the fire!—To her utter astonishment, "the *young lady*" confessed that she had her "*long busk*" on,—that her "*lacing*," which was excessively tight, was in a "hard knot," and that she "could not possibly stoop" to put on the kettle! On another occasion, the writer was obliged to stop and admire one of those faithful imitators of high life, who, attired in a rich yellow barege frock, with gorgeous balloon sleeves, and *laced* to a most fashionable degree, was occupied in sweeping out one of the filthiest gutters in Seventh Street! Nothing was wanting to complete the picture, but one of the exquisitely dressed and Russian belted "gemmen," we occasionally see in the streets, to have shaded her with an umbrella, while she was engaged in discharging this receptacle of "liquid sweets."

speak of, an excess of false delicacy under such circumstances has become fashionable. If all the rest of the world were to resolve on the use of tight lacing, mothers should determine to lay it aside, if only in compassion to their offspring, whose health and happiness may otherwise be entirely sacrificed. If we make strict examination among children of *fashionable* parents, we shall find proof sufficient of this, even if nothing worse be discovered than pale, delicate, rickety, or scrofulous subjects, whose appearance proclaims imperfect health with enfeebled and easily injured constitutions. The injuries produced on many delicate females by tight lacing, before and after marriage, have been sufficiently great, in numerous instances, to destroy all the joyous hopes and anticipations which are incident to maternity, and rendered the conjugal condition one of unceasing disappointment and gloomy solitude.

Enough, however, has been said on this subject, although we have given but an imperfect catalogue of the mischiefs produced by tight lacing. Much of what we have said will be regarded by tight lacers as a mere attempt to alarm, because they have not yet especially suffered from this cause. If inquiry be made of physicians residing in our cities, ample confirmation of all we have stated may be obtained, and proofs of still greater evils from this cause afforded. We cannot, however, hope to effect much against the preponderating influence of fashion, considering how often it has been attempted by others unsuccessfully. Nevertheless, we have esteemed it a duty to make even this imperfect essay, hoping that possibly *one* parent might be convinced, or one female saved from injury."*

---

### XXXVI.

### Mr. Jewel on Nitrate of Silver in Leucorrhœa.

* Boston Med. and Surg. Journal.

